# ^18^F-FAPI PET/CT performs better in evaluating mediastinal and hilar lymph nodes in patients with lung cancer: comparison with ^18^F-FDG PET/CT

**DOI:** 10.1186/s40001-023-01494-9

**Published:** 2024-01-03

**Authors:** Yuyun Sun, Yun Sun, Zili Li, Shaoli Song, Kailiang Wu, Jingfang Mao, Jingyi Cheng

**Affiliations:** 1 Department of Nuclear Medicine, Shanghai Proton and Heavy Ion Center, Fudan University Cancer Hospital, 4365 Kangxin Road, Shanghai, 201321 China; 2 Shanghai Key Laboratory of Radiation Oncology (20dz2261000), Shanghai, China; 3 Shanghai Engineering Research Center of Proton and Heavy Ion Radiation Therapy, Shanghai, China; 4 Department of Radiotherapy, Shanghai Proton and Heavy Ion Center, Fudan University Cancer Hospital, Shanghai, 201321 China; 5 Department of Nuclear Medicine, Shanghai Proton and Heavy Ion Center, Shanghai, 201321 China

**Keywords:** ^18^F-FAPI PET/CT, Mediastinal and hilar lymph node, Lung cancer, ^18^F-FDG PET/CT

## Abstract

**Background:**

The aim of this study was to evaluate the efficacy of fluorine 18 (^18^F) labeled fibroblast activation protein inhibitor (FAPI) in identifying mediastinal and hilar lymph node metastases and to develop a model to quantitatively and repeatedly identify lymph node status.

**Methods:**

Twenty-seven patients with 137 lymph nodes were identified by two PET/CT images. The sensitivity, specificity, accuracy, positive predictive value (PPV), and negative predictive value (NPV) of lymph node status were analyzed, and the optimal cut-off value was identified by ROC analysis.

**Results:**

The SUVmax of metastatic lymph nodes on ^18^F-FAPI was higher than that on ^18^F-FDG PET/CT (10.87 ± 7.29 vs 6.08 ± 5.37, *p* < 0.001). ^18^F-FAPI presented much greater lymph node detection sensitivity, specificity, accuracy, PPV and NPV than ^18^F-FDG PET/CT (84% vs. 71%; 92% vs. 67%; 90% vs. 69%, 84% vs. 52%, and 92% vs. 83%, respectively). Additionally, the diagnostic effectiveness of ^18^F-FAPI in small lymph nodes was greater than that of ^18^F-FDG PET/CT (specificity: 96% vs. 72%; accuracy: 93% vs. 73%; PPV: 77% vs. 33%, respectively). Notably, the optimal cut-off value for specificity and PPV of ^18^F-FAPI SUVmax was 5.3; the optimal cut-off value for sensitivity and NPV was 2.5.

**Conclusion:**

^18^F-FAPI showed promising diagnostic efficacy in metastatic mediastinal and hilar lymph nodes from lung cancer patients, with a higher SUVmax, especially in small metastatic nodes, compared with ^18^F-FDG. In addition, this exploratory work recommended optimal SUVmax cutoff values to distinguish between nonmetastatic and metastatic lymph nodes, thereby advancing the development of image-guided radiation.

*Trial registration* ClinicalTrials.gov identifier: ChiCTR2000036091.

## Background

Lung cancer is the oncologic disease with the highest mortality rate worldwide, and it accounts for over 20% of cancer-related deaths each year [1]. For patients with locally advanced lung cancer, regional lymph node staging is very important, as it guides the choice of treatment. Histopathological and imaging examinations are the most commonly used methods to predict mediastinal and hilar lymph node staging in patients with lung cancer.

Endobronchial ultrasound-guided transbronchial needle aspiration (EBUS-TBNA) is the “gold standard” for the evaluation of mediastinal and hilar lymph nodes in patients with lung cancer and is recommended by the National Comprehensive Cancer Network [2]; however, the diagnostic accuracy of EBUS-TBNA is insufficient due to intratumor heterogeneity and endoscopist skills [3, 4]. On the other hand, enhanced computed tomography (CT) is a commonly used imaging technique for evaluating mediastinal and hilar lymph nodes in patients with lung cancer, and a cutoff value of 10 mm for the short-axis diameter has been suggested to define abnormal lymph nodes [5, 6]. However, a retrospective study involving 2817 mediastinal and hilar lymph nodes revealed that the sensitivities of enhanced CT in diagnosing mediastinal and hilar lymph nodes were only 18.9% and 17.0%, respectively [7]. ^18^F-fluorodeoxyglucose ([^18^F]-FDG) positron emission tomography/computed tomography (PET/CT) is always recommended for tumor diagnosis and staging [8], but it is not sufficiently specific for diagnosing lymph nodes, as inflammatory lesions also demonstrate enhanced FDG uptake [9, 10].

^68^Ga/^18^F-labeled fibroblast-activation protein inhibitor (FAPI) PET/CT has been used in various kinds of tumors and demonstrated a complementary role in discriminating malignant from benign lesions [11], and it can reveal more metastatic lymph nodes in various cancers than ^18^F-FDG [12–15]. A recent case report demonstrated that ^68^Ga-FAPI PET/CT scans downstage the TNM stage of squamous cell lung cancer due to the lack of FAPI uptake in the enlarged right lower paratracheal lymph node [10]. These encouraging results prompted us to compare ^18^F-AlF-FAPI-04 (^18^F-FAPI) PET/CT to ^18^F-FDG PET/CT for identifying mediastinal and hilar lymph nodes in lung cancer patients.

This study aims to compare the diagnostic efficacy of ^18^F-FAPI PET/CT and ^18^F-FDG PET/CT in patients with locally advanced lung cancer for the diagnosis of metastatic mediastinal and hilar lymph nodes. More importantly, an optimal SUVmax cutoff value should be determined to quantitatively and frequently diagnoses lymph nodes, especially in small lymph nodes.

## Methods

### Study participants

Twenty-seven patients diagnosed with lung cancer referred to staging of disease were prospectively recruited and underwent both ^18^F-FDG PET/CT and ^18^F-FAPI PET/CT scans. The patients were enrolled as part of a larger ongoing study in our institution to evaluate the role of ^18^F-FAPI PET/CT in the imaging of mediastinal and hilar lymph node metastasis in lung cancer patients (ClinicalTrials.gov identifier: ChiCTR2000036091). The inclusion criteria were as follows: (i) patients diagnosed with lung cancer pathologically; (ii) paired ^18^F-FDG and ^18^F-FAPI PET/CT were performed within one week. The exclusion criteria were (i) pregnant patients, (ii) patients with another malignant disease, and (iii) participants who were unwilling to undergo ^18^F-FDG or ^18^F-FAPI PET/CT examination. This study was approved by the institutional review board (IRB) of the Shanghai Proton and Heavy Ion Center (SPHIC) (ethical code: 2106-49-01) and conducted in accordance with the 1964 Declaration of Helsinki and its later amendments or comparable ethical standards, and all subjects signed an informed consent form. The final diagnosis was confirmed by EBUS or follow-up. Follow-up contrast-enhanced CT was performed 6 months after treatment. Lymph nodes in the follow-up images were considered malignant based on the following criteria: (i) the morphology, growth pattern and enhancement pattern of the nodes were consistent with the characteristics of malignant tumors, (ii) tumor progression after treatment, and (iii) tumor shrinkage after treatment.

### Radiopharmaceuticals and PET/CT imaging

The synthesis of [^18^F]-FDG was based on a cyclotron (Siemens CTI RDS Eclips ST, Knoxville, TN). ^18^F-AlF-FAPI-04 preparation was performed according to a published procedure [16]. The radiochemical purity was over 95%.

Paired ^18^F-FDG and ^18^F-FAPI scans were performed within 7 days. For ^18^F-FDG PET/CT, patients were required to fast for at least 6 h, and their blood glucose level had to be under 11.1 mmol/L. For ^18^F-FAPI PET/CT, no specific preparation was requested. The imaging procedures were carried out on a Biography 16 PET/CT scanner (Siemens Healthcare, Erlangen, Germany) one hour after ^18^F-FDG (~ 0.1 mCi/kg, 370 MBq/kg) or ^18^F-FAPI (~ 0.1 mCi/kg, 370 MBq/kg) injection. PET image datasets were iteratively reconstructed using an ordered-subset expectation maximization iterative reconstruction by applying CT data for attenuation correction.

### Image interpretation

Two experienced nuclear medicine physicians analyzed and interpreted the images in a blinded manner, and in cases of disagreement, a consensus was reached. SUVmax for each lymph node was calculated by placing a spheroid-shaped volume of interest within the node on a multimodality computer platform (Syngo, Siemens, Knoxville, Tennessee, USA). Positive ^18^F-FDG and ^18^F-FAPI uptake was defined as focal avidity greater than the background of the mediastinal blood pool. Lymph nodes were assessed using enhanced CT scans, and nodes with short-axis diameters less than 10 mm were defined as small lymph nodes; otherwise, they were defined as large lymph nodes.

### Statistical analyses

Data were analyzed by SPSS statistical software (version 25.0, SPSS, IBM Inc., New York, USA). Demographic data and PET/CT parameters were summarized as the mean with standard deviation or frequencies with percentages. The Wilcoxon signed-rank test was used to analyze the differences between PET/CT parameters. P < 0.05 was considered statistically significant, and all analyses were two sided.

## Results

### Patient characteristics

**Fig. 1:**
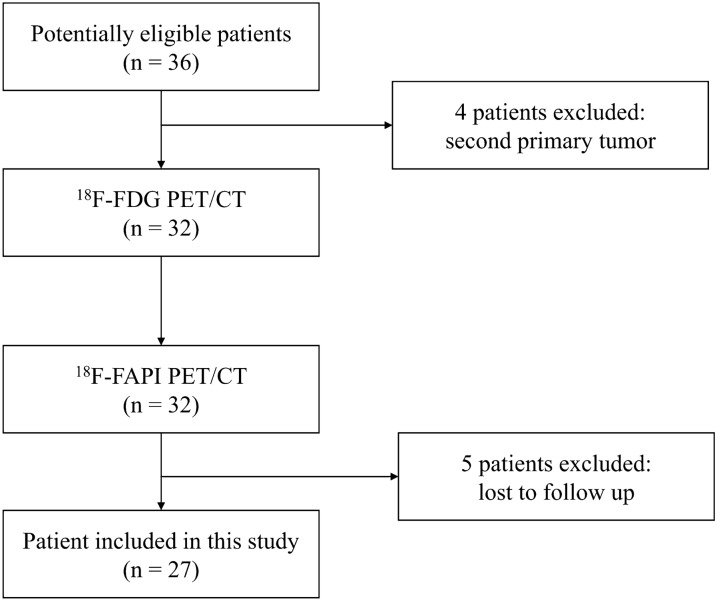
Flow diagram shows participant selection details. *FAPI*  fibroblast activation protein inhibitor, *FDG* fluorodeoxyglucose, ^18^F = fluorine 18

A total of 36 patients with locally advanced lung cancer were registered in the study, and they underwent PET/CT for tumor staging. Four patients had a second primary tumor and 5 patients missed in the follow-up period; these patients were excluded from the study (Fig. 1). Finally, 27 patients with 137 mediastinal and hilar lymph nodes were included. Of the 137 lymph nodes, 106 were mediastinal lymph nodes and 31 were hilar lymph nodes. The clinical characteristics of these patients are shown in Table 1.

**Table 1:** Patient characteristics (*n* = 27)

Characteristics	Value
Age(year)	58 ± 7
Sex	
Male	18
Female	9
T stage	
T1	2
T2	11
T3	14
T4	0
N stage	
N0	0
N1	3
N2	13
N3	11

In the evaluation of the lymph nodes, histopathological examination or contrast-enhanced CT was used. Fourteen nodes in 6 patients were confirmed with pathologic examination, and 123 nodes in 21 patients were confirmed with contrast-enhanced CT before treatment and the follow-up enhanced CT images. Finally, forty-five out of 137 lymph nodes were found to be cancerous, including 32 mediastinal and 13 hilar lymph nodes. In addition, 85 lymph nodes (62%, 85/137) with a short-axis diameter less than 10 mm were defined as small lymph nodes, including 13 metastatic and 72 nonmetastatic lymph nodes.

### Improved metastatic lymph node detection with ^18^F-FAPI PET/CT

**Fig. 2:**
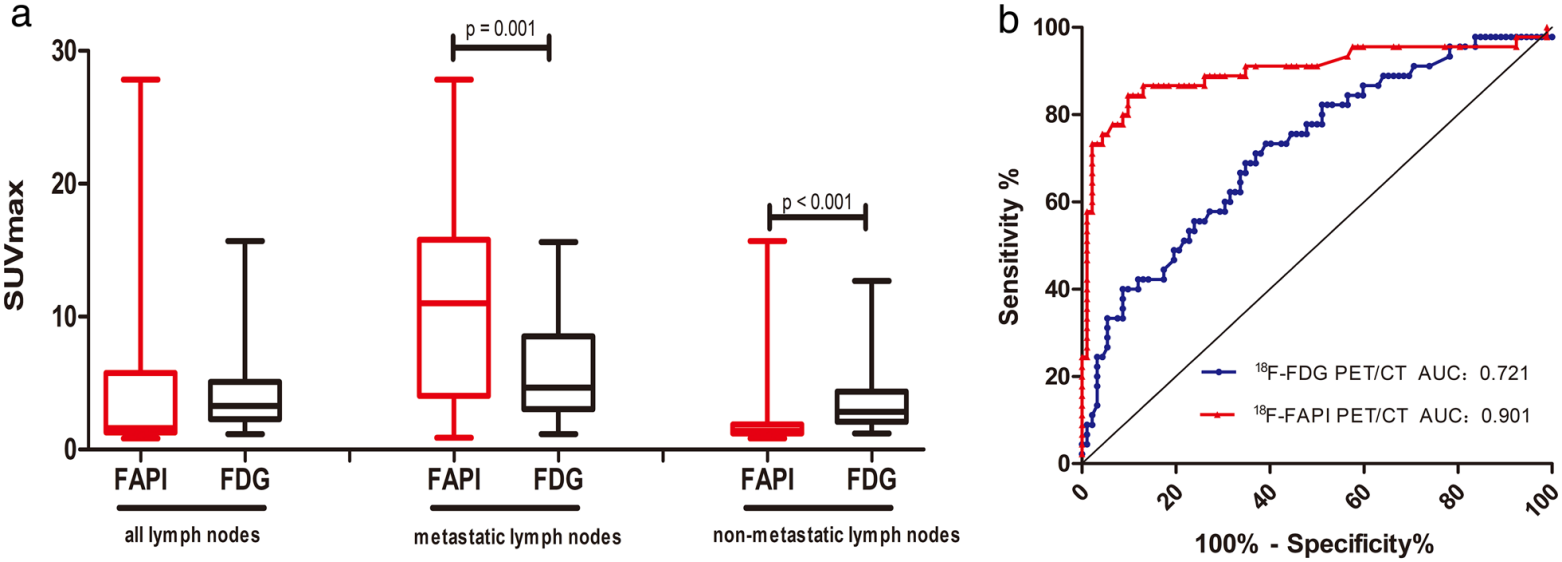
Comparison of the performance of ^18^F-FAPI and.^18^F-FDG PET/CT in diagnosing mediastinal and hilar lymph nodes. Comparison of the tracer uptakes **a** and ROC analysis of SUVmax derived from the two PET/CTs for identifying lymph nodes status **b**

In all 137 lymph nodes, the ^18^F-FAPI-derived SUVmax was 4.80 ± 6.11, and the ^18^F-FDG-derived SUVmax was 4.33 ± 3.69, *p* = 0.414. In all 45 metastatic lymph nodes, the mean value of SUVmax on ^18^F-FAPI PET/CT images was 10.87 ± 7.29, and SUVmax on ^18^F-FDG PET/CT images was 6.08 ± 5.37, *p* = 0.001, while in 92 benign lymph nodes, the SUVmax of ^68^ Ga-FAPI PET/CT was 1.87 ± 1.82, and the SUVmax of ^18^F-FDG PET/CT was 3.47 ± 2.04, *p* < 0.001. In summary, ^18^F-FAPI PET/CT showed a much higher SUVmax value than ^18^F-FDG PET/CT in metastatic lymph nodes but a much lower SUVmax value than ^18^F-FDG in benign lymph nodes. Moreover, in ^18^F-FDG PET/CT, the SUVmax values in metastatic and nonmetastatic lymph nodes presented substantial overlap, although the *p*-value was less than 0.05. However, in ^18^F-FAPI PET/CT, there was no overlap, and the *p* value was less than 0.001, Fig. 2a.

**Fig. 3:**
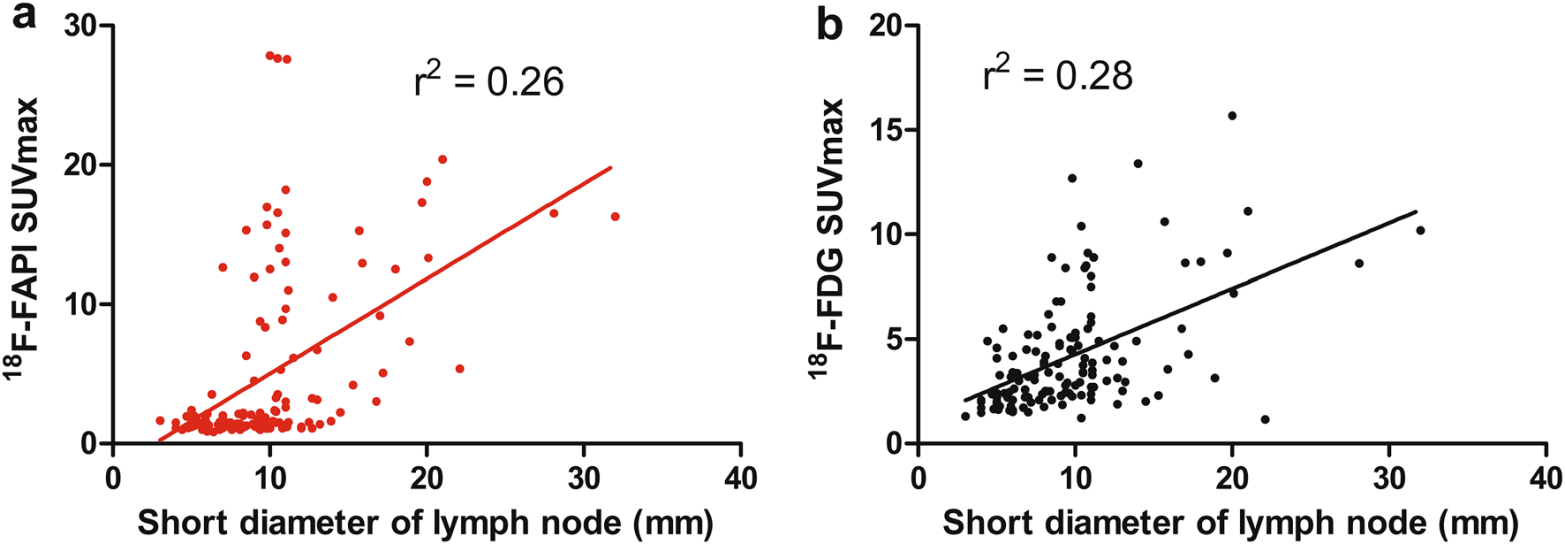
The correction between short diameter of lymph nodes and SUVmax. The correction between short diameter of lymph nodes and ^18^F-FAPI SUVmax **a** and.^18^F-FDG PAPI SUVmax **b**

In addition to the correction between SUVmax and the nature of lymph nodes, the relationship between SUVmax and the diameters of these nodes were compared. However, neither SUVmax of ^18^F-FAPI nor SUVmax of ^18^F-FDG had significant correction with the size of nodes (*r*^2^ = 0.26 and 0.28, respectively), Fig. 3. In summary, there was a difference between SUVmax of ^18^F-FAPI and the nature of lymph nodes, but no relationship in SUVmax and lymph node size.

Additionally, ^18^F-FAPI was a superior method in evaluating mediastinal hilar lymph nodes, with a larger area under the receiver operating characteristic (ROC) curve comparing with ^18^F-FDG (0.901 vs. 0.721). The optimal cutoff value for diagnosis of ^18^F-FAPI and ^18^F-FDG PET/CT was 2.5 and 3.4 by ROC analysis, respectively (Fig. 2b). On ^18^F-FAPI PET/CT, the sensitivity, specificity, accuracy, positive predictive value, and negative predictive value were 84%, 92%, 90%, 84%, and 92%, respectively. In ^18^F-FDG PET/CT imaging, the sensitivity, specificity, accuracy, positive predictive value, and negative predictive value were 71%, 67%, 69%, 52%, and 83%, respectively (Table 2).

**Table 2:** Comparison of ^18^F-FAPI and ^18^F-FDG PET/CT in the evaluation of all the mediastinal and hilar lymph nodes

	^18^F-FAPI	^18^F-FDG	*P* value
True positive (No. of lymph nodes)	38	32	/
False positive (No. of lymph nodes)	7	30	/
False negative (No. of lymph nodes)	7	13	/
True negative (No. of lymph nodes)	85	62	/
Sensitivity (%)	84 [71, 93]	71 [57, 82]	0.031*
Specificity (%)	92 [85, 97]	67 [57, 76]	<0 .001*
Accuracy (%)	90 [83, 94]	69 [60, 76]	<0 .001*
PPV (%)	84 [71, 93]	52 [39, 64]	/
NPV (%)	92 [85, 97]	83 [72, 90]	/

Data in brackets are 95% CIs. FAPI = fibroblast activation protein inhibitor, *FDG *fluorodeoxyglucose, ^18^F = fluorine 18, *PPV* positive predictive value, *NPV* negative predictive value

^*^Indicates statistical significance

### ^18^F-FAPI PET/CT improves the diagnostic accuracy for small metastatic lymph nodes

**Fig. 4:**
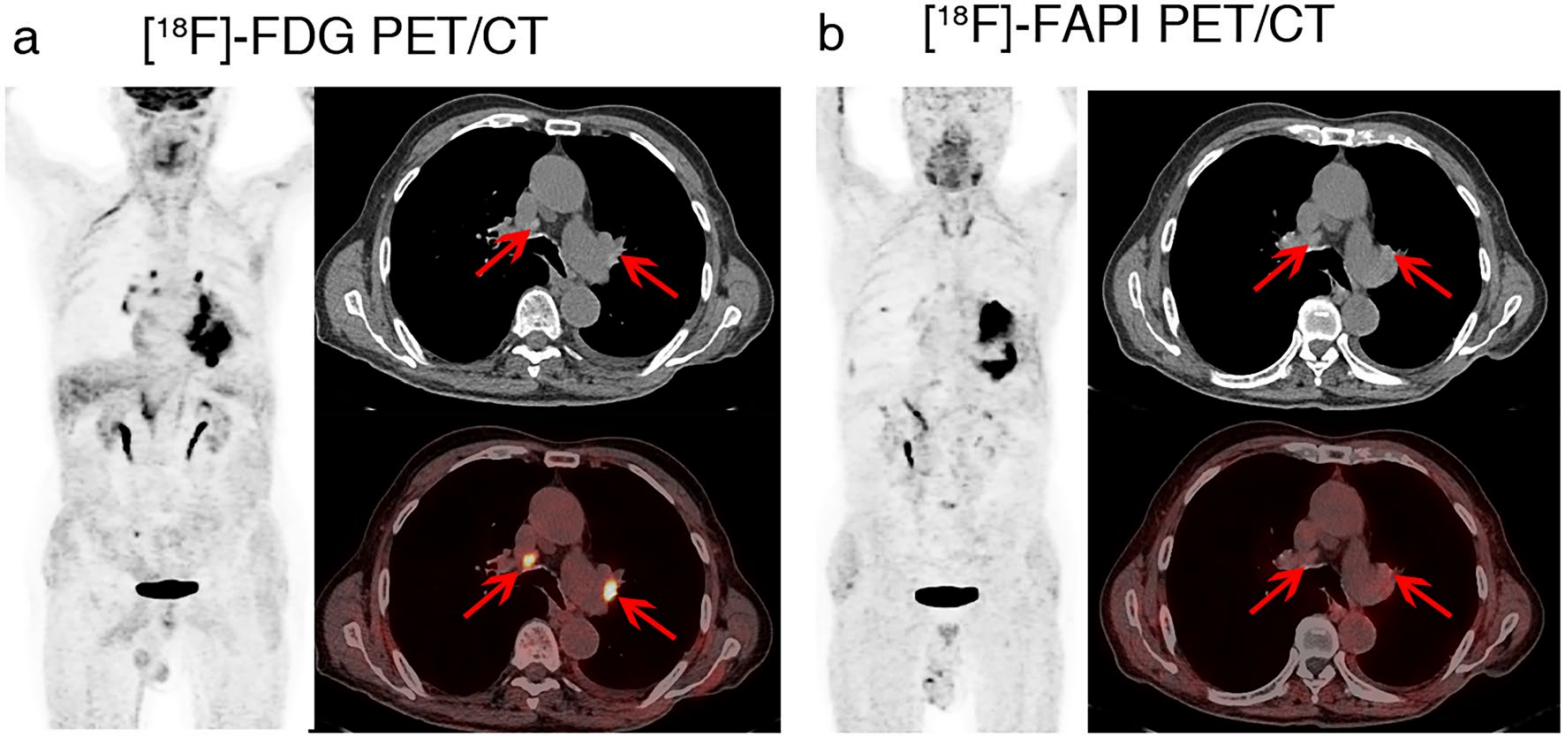
A 79-year-old man with a diagnosis of lung cancer. Lymph nodes in region 4R and 10L with intense ^18^F-FDG uptake (**a**, red arrows) and no ^18^F-FAPI uptake (**b**, red arrows) were confirmed as nonmetastatic lymph nodes pathologically

**Fig. 5:**
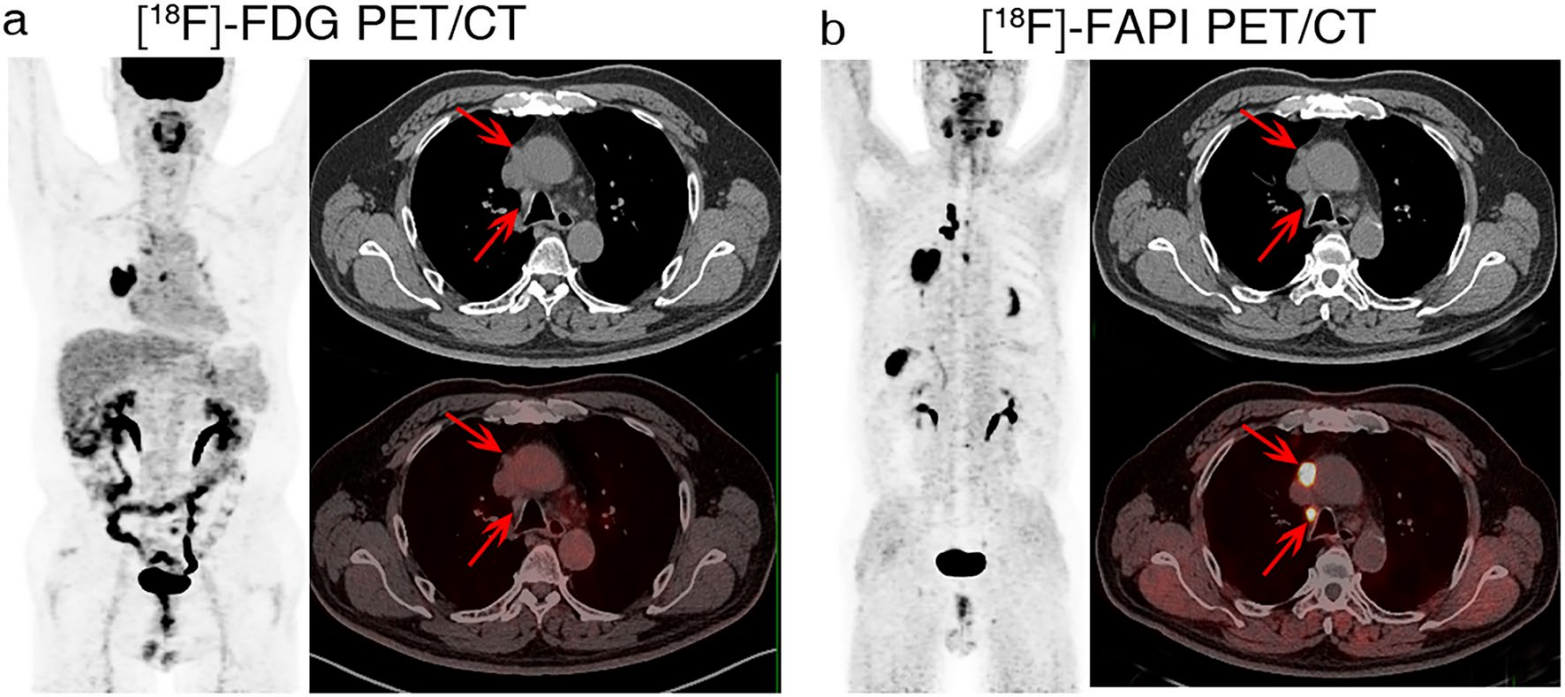
A 58-year-old man with a diagnosis of lung cancer. Small (0.7 * 0.7 cm) metastatic lymph nodes in region 4R showed no ^18^F-FDG-avidity (**a**, red arrows), but remarkable.^18^F-FAPI-avidity (**b**, red arrows)

When small LNs were considered, ^18^F-FAPI performed much better than ^18^F-FDG PET/CT. A total of 85 lymph nodes with a short-axis diameter less than 10 mm were confirmed to be small lymph nodes, including 13 metastatic and 72 nonmetastatic nodes. In ^18^F-FAPI PET/CT, the SUVmax of small metastatic lymph nodes was substantially higher than that of small nonmetastatic lymph nodes (9.29 ± 7.57 vs. 1.67 ± 1.73, *p* = 0.001). Similarly, the SUVmax based on ^18^F-FDG was greater in small metastatic lymph nodes than in small nonmetastatic nodes (4.37 ± 1.85 vs. 3.10 ± 1.86, *p* = 0.001). As a result, ^18^F-FAPI presented much higher specificity, accuracy, and positive prediction value (PPV) than ^18^F-FDG PET/CT (specificity: 96% vs. 72%; accuracy: 93% vs. 73%; PPV: 77% vs. 33%, respectively). In ^18^F-FDG PET, false-positives were observed in 20 patients due to inflammation. However, in ^18^F-FAPI PET, only 3 false-positives were observed. The diagnostic efficacies of the two procedures are compared in Table 3. A typical case showing false-positive ^18^F-FDG uptake and true-negative ^18^F-FAPI uptake in mediastinal and hilar lymph nodes is shown in Fig. 4, and a representative case displaying intense ^18^F-FAPI but negative ^18^F-FDG uptake in small metastatic lymph nodes is shown in Fig. 5.

**Table 3:** Comparison of ^18^F-FAPI and ^18^F-FDG in the evaluation of small mediastinal and hilar lymph nodes

	^18^F-FAPI	^18^F-FDG	*P* value
True positive (No. of lymph nodes)	10	10	/
False positive (No. of lymph nodes)	3	20	/
False negative (No. of lymph nodes)	3	3	/
True negative (No. of lymph nodes)	69	52	/
Sensitivity (%)	77 [49, 93]	77 [49, 93]	NS
Specificity (%)	96 [88, 99]	72 [61, 81]	< .001*
Accuracy (%)	93 [85, 97]	73 [63, 81]	< .001*
PPV (%)	77 [49, 93]	33 [19, 51]	/
NPV (%)	96 [88, 99]	95 [84, 98]	/

Data in brackets are 95% CIs. *FAPI* fibroblast activation protein inhibitor, FDG = fluorodeoxyglucose, ^*18*^*F*  fluorine 18, *PPV* positive predictive value, *NPV* negative predictive value, *NS*  no statistics significance

^*^Indicates statistical significance

### SUVmax cut-off values for differentiating metastatic from benign lymph nodes

**Fig. 6:**
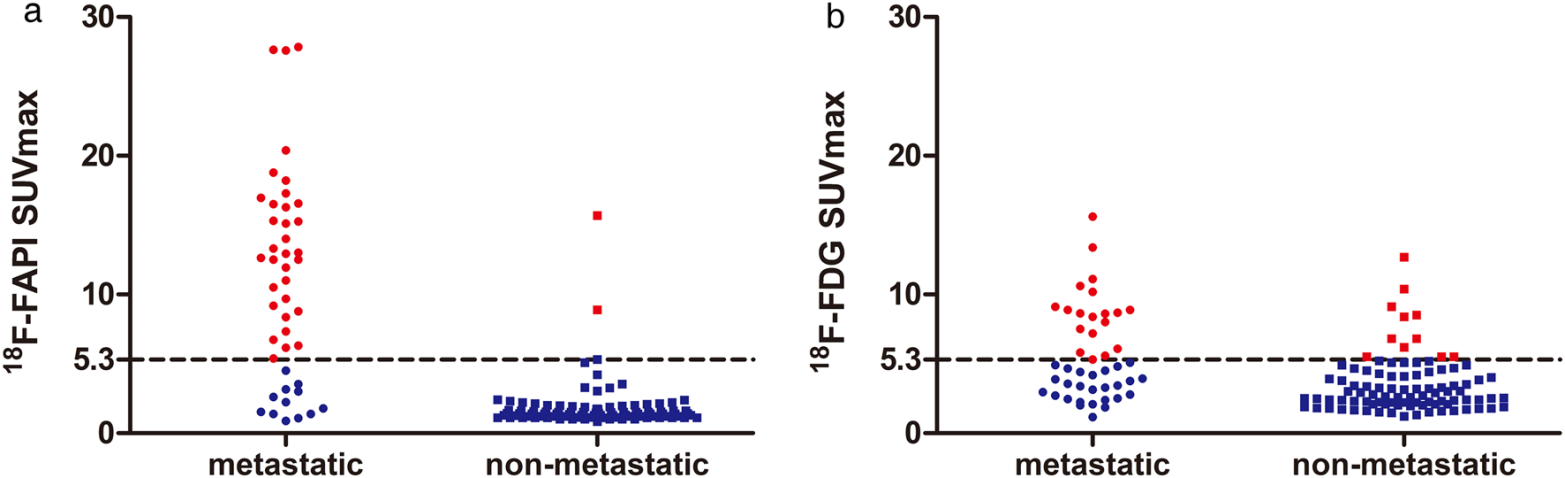
The SUVmax of every lymph node and the optimal cut-off value for the evaluation of metastatic and nonmetastatic lymph nodes. 98% (90/92) nonmetastatic nodes had a ^18^F-FAPI-derived SUVmax less than 5.3 and 73% (33 of 45) metastatic nodes showed a ^18^F-FAPI-derived SUVmax larger than 5.3 **a**. The SUVmax of metastatic nodes derived from.^18^F-FDG overlapped that of nonmetastatic nodes **b**

Figure 6 demonstrates the distribution of SUVmax in metastatic and nonmetastatic lymph nodes. In the nonmetastatic group, 90 lymph nodes had the ^18^F-FAPI SUVmax smaller than 5.3, and only 2 nodes had SUVmax larger than 5.3, resulting in a high specificity (98%). As a result, we evaluated the diagnostic performance of ^18^F-FAPI when choosing 5.3 as a cut-off value. The sensitivity, specificity, accuracy, positive predictive value, and negative predictive value were 73%, 98%, 90%, 94%, and 88%, respectively. Additionally, mean value is commonly used in data analysis, so we investigated the diagnostic performance when SUVmax was 3.5, which was the mean SUVmax value (Table 4). As aforementioned, 2.5 was also an optimal cutoff value for diagnosis, so we compared the diagnostic performance when the cutoff values were 5.3, 3.5 and 2.5 (Table 4). All the three values demonstrated similar diagnostic accuracy, but when the cut-off value was 5.3, the ^18^F-FAPI SUVmax could improve specificity and PPV. When the cutoff value was 2.5, SUVmax could improve sensitivity and NPV.

**Table 4:** Comparison of diagnostic value between different cut-off values in ^18^F-FAPI PET/CT

Cut-off value	Sensitivity (%)	Specificity (%)	Accuracy (%)	PPV (%)	NPV (%)
5.3	73 [59, 84]	98 [92, 99]	90 [83, 94]	94 [81, 99]	88 [80, 94]
3.5	78 [63, 89]	93 [86, 97]	88 [82, 93]	85 [71, 94]	90 [82, 95]
2.5	84 [71, 93]	92 [85, 97]	90 [83, 94]	84 [71, 93]	92 [85, 97]

Data in brackets are 95% CIs. FAPI = fibroblast activation protein inhibitor, ^*18*^*F* fluorine 18, *PPV*  positive predictive value, *NPV* negative predictive value

In FDG PET/CT, the values of 3.5 and 5.3 were inapplicable, because the SUVmax of nonmetastatic lymph nodes overlapped with that of metastatic lymph nodes.

## Discussion

The present study indicated that ^18^F-FAPI PET/CT had much higher diagnostic efficacy in mediastinal and hilar lymph nodes in patients with lung cancer, especially in small metastatic lymph nodes (size less than 10 mm), when compared with ^18^F-FDG. Additionally, two cutoff values were identified in this study. The optimal cut-off value for specificity and PPV of ^18^F-FAPI SUVmax was 5.3; the optimal cut-off value for sensitivity and NPV was 2.5; this result means that a noninvasive and accurate diagnostic model could be established with ^18^F-FAPI PET/CT.

A study has shown that intrathoracic nodal status is considered to be positive for metastatic spread if the FDG activity of the node is higher than the mediastinal background [17]; however, a high level of FDG uptake can also be noted in various benign conditions, such as different infective/inflammatory processes [18, 19]. A previous study indicated that a benign mediastinal lymph node with 34.5 mm in size was FDG-avid [20]. In the present study, metastatic lymph nodes had significantly higher FAPI-derived SUVmax than nonmetastatic lymph nodes, but in ^18^F-FDG PET/CT imaging, although metastatic lymph nodes showed higher uptake than nonmetastatic nodes, the SUVmax in metastatic lymph nodes exhibited substantial overlap that in nonmetastatic lymph nodes. This finding suggested that ^18^F-FAPI PET/CT may help address the limitation of ^18^F-FDG in diagnosing lymph nodes and expand the clinical indications of PET/CT. In addition, our study showed that ^18^F-FAPI PET/CT was more sensitive and specific than ^18^F-FDG in diagnosing mediastinal and hilar lymph nodes, consistent with previous studies [21]. The variable positive predictive rate for the assessment of mediastinal lymph node involvement with ^18^F-FDG PET/CT has also been recognized, ranging from 32.3% to 89% [22, 23]. The low and dispersive predictive rate limits the application of ^18^F-FDG in the assessment of mediastinal lymph nodes, and our results suggest that ^18^F-FAPI PET/CT might be an alternative imaging technique.

Additionally, according to ACCP evidenced-based clinical practice guidelines, lymph nodes with short-axis diameters larger than 10 mm were considered abnormal lymph nodes [6]. As a result, in the present study, lymph nodes with short-axis diameters less than 10 mm were defined as small lymph nodes. The size of the lymph nodes was an important factor affecting the diagnostic efficiency on imaging scans, including CT and PET/CT scans. For very small lesions (< 10 mm), visibility is low due to the partial volume effect and low tumor metabolic activity [24, 25]. However, previous studies indicated that FAPI PET/CT was better than FDG PET/CT in diagnosing metastatic lymph nodes. A recent study indicated that ^68^ Ga-FAPI PET/CT had a diagnostic accuracy of 100% in diagnosing metastatic lymph nodes from non-small cell lung cancer, while ^18^F-FDG had an accuracy of 30% [26]. Lijuan Wang et al. showed that ^68^ Ga-FAPI detected 356 lymph nodes, while ^18^F-FDG PET/CT detected 320 nodes, and 22 among the 36 lymph nodes detected only by FAPI PET/CT had a diameter shorter than 10 mm [27]. In the present study, the number of small positive lymph nodes detected by the two PET/CTs was the same, but the specificity and accuracy for diagnosing small lymph nodes were higher for ^18^F-FAPI than for ^18^F-FDG. Previous studies indicated that in the earlier stage of metastatic lymph node, the number of tumor cells was small and could not be detected by ^18^F-FDG [28, 29]. Before tumor cells are located in the lymph nodes, they secrete some factors to promote premetastatic lymph nodes by activating fibroblast reticular cells, which differentiate into CAFs and express high levels of FAP [30–32]. This may explain why ^18^F-FAPI PET/CT is better in detecting small metastatic lymph nodes.

Another important finding of this study is the diagnostic cut-off values. As the pathologic gold standard, the sensitivity of EBUS in mediastinal staging for lung cancer ranges from 45 to 93%, and the NPV was 93% [33]. In this study, using SUVmax 2.5 as the cut-off value, the sensitivity was 84% and the NPV was 92%, which was similar to that of the EBUS method. When the cut-off was 5.3, the specificity and PPV were highly increased. To the best of our knowledge, cut-off values for SUVmax to identify lymph node status have not been investigated previously. Such a reference value may provide a simple, powerful and repeatable clinical tool for the prediction of lymph nodes in patients with lung cancer.

This systematic study investigated the value of ^18^F-FAPI PET/CT in diagnosing mediastinal and hilar lymph nodes in patients with lung cancer and obtained two optimal values for discriminating metastatic from benign nodes. Our results indicated the value of ^18^F-FAPI PET/CT in the detection of mediastinal and hilar lymph nodes secondary to lung cancer. However, there were some limitations in this study. First, this was a single-center study with a small sample size. Second, not all lymph nodes were diagnosed by pathology due to the difficulty of obtaining tissue samples. However, the results were obtained with enhanced CT and follow-up.

## Conclusion

^18^F-FAPI showed promising diagnostic efficiency in metastatic mediastinal and hilar lymph nodes from lung cancer patients with a better sensitivity and negative predictive value, particularly in small nodes (size less than 10 mm), than ^18^F-FDG. Additionally, this preliminary study proposed optimal cutoff values of 5.3 to distinguish nonmetastatic from metastatic nodes, which could obtain a similar sensitivity of 94.29% with EBUS and a much higher NPV of nonmetastatic LNs.

## Data Availability

The datasets generated and analyzed during the current study are available from the corresponding author on reasonable request.

## Abbreviations

FAP: Fibroblast activation protein
FAPI: Fibroblast activation protein inhibitor
18F-FDG: Fluorine 18 fluorodeoxyglucose
18F-FAPI: Fluorine 18-labeled fibroblast activation protein inhibitor
SUVmax: Maximum standardized uptake value
PPV: Positive predictive value
NPV: Negative predictive value

## References

[1] Oliver AL (2022). Lung cancer: epidemiology and screening. Surg Clin North Am.

[2] Ettinger DS, Wood DE, Akerley W, Bazhenova LA, Borghaei H, Camidge DR (2015). Non-small cell lung cancer version 62015. J Natl Compr Canc Netw.

[3] Jalil BA, Yasufuku K, Khan AM (2015). Uses, limitations, and complications of endobronchial ultrasound. Proc (Bayl Univ Med Cent).

[4] Schmid-Bindert G, Jiang H, Kähler G, Saur J, Henzler T, Wang H (2012). Predicting malignancy in mediastinal lymph nodes by endobronchial ultrasound: a new ultrasound scoring system. Respirology.

[5] Silvestri GA, Tanoue LT, Margolis ML, Barker J, Detterbeck F (2003). The noninvasive staging of non-small cell lung cancer: the guidelines. Chest.

[6] Silvestri GA, Gould MK, Margolis ML, Tanoue LT, McCrory D, Toloza E (2007). Noninvasive staging of non-small cell lung cancer: ACCP evidenced-based clinical practice guidelines. Chest.

[7] Fréchet B, Kazakov J, Thiffault V, Ferraro P, Liberman M (2018). Diagnostic accuracy of mediastinal lymph node staging techniques in the preoperative assessment of nonsmall cell lung cancer patients. J Bronchology Interv Pulmonol.

[8] Kandathil A, Kay FU, Butt YM, Wachsmann JW, Subramaniam RM (2018). Role of FDG PET/CT in the of TNM staging of non-small cell lung cancer. Radiographics.

[9] González-Cruz C, Bodet D, Muñoz-Couselo E, García-Patos V (2021). Mediastinal FDG-positive lymph nodes simulating melanoma progression: drug-induced sarcoidosis like/lymphadenopathy related to ipilimumab. BMJ Case Rep.

[10] Shang Q, Zhao L, Pang Y, Meng T, Chen H (2022). Differentiation of reactive lymph nodes and tumor metastatic lymph nodes With 68Ga-FAPI PET/CT in a patient with squamous cell lung cancer. Clin Nucl Med.

[11] Guglielmo P, Guerra L (2021). Radiolabeled fibroblast activation protein inhibitor (FAPI) PET in oncology: has the time come for 18F-fluorodeoxyglucose to think to a well-deserved retirement?. ClinTransl Imaging.

[12] Çermik TF, Ergül N, Yılmaz B, Mercanoğlu G (2022). Tumor imaging with 68Ga-DOTA-FAPI-04 PET/CT: comparison with 18F-FDG PET/CT in 22 different cancer types. Clin Nucl Med.

[13] Kratochwil C, Flechsig P, Lindner T, Abderrahim L, Altmann A, Mier W (2019). (68)Ga-FAPI PET/CT: tracer uptake in 28 different kinds of cancer. J Nucl Med.

[14] Giesel FL, Adeberg S, Syed M, Lindner T, Jiménez-Franco LD, Mavriopoulou E (2021). FAPI-74 PET/CT using either (18)F-AlF or Cold-Kit (68)Ga labeling: biodistribution, radiation dosimetry, and tumor delineation in lung cancer patients. J Nucl Med.

[15] Pang Y, Zhao L (2021). Comparison of (68)Ga-FAPI and (18)F-FDG uptake in gastric. Duodenal Colorectal Cancers.

[16] Kou Y, Jiang X, Yao Y, Shen J, Jiang X, Chen S (2022). Physiological tracer distribution and benign lesion incidental uptake of Al18F-NOTA-FAPI-04 on PET/CT imaging. Nucl Med Commun.

[17] Gunluoglu MZ, Melek H, Medetoglu B, Demir A, Kara HV, Dincer SI (2011). The validity of preoperative lymph node staging guidelines of European society of thoracic surgeons in non-small-cell lung cancer patients. Eur J Cardiothorac Surg.

[18] Ding RL, Cao HY, Hu Y, Shang CL, Xie F, Zhang ZH (2017). Lymph node tuberculosis mimicking malignancy on (18)F-FDG PET/CT in two patients: a case report. Exp Ther Med.

[19] Maccarone MT (2019). FDG-PET Scan in sarcoidosis: clinical and imaging indications. Curr Med Imaging Rev.

[20] T Fujiwara T Nakajima. The combination of endobronchial elastography and sonographic findings during endobronchial ultrasound-guided transbronchial needle aspiration for predicting nodal metastasis. 2019;10:2000-5. 10.1111/1759-7714.13186 10.1111/1759-7714.13186 PMC6775026 31474004

[21] Qin C, Shao F, Gai Y, Liu Q, Ruan W, Liu F (2022). (68)Ga-DOTA-FAPI-04 PET/MR in the evaluation of gastric carcinomas: comparison with (18)F-FDG PET/CT. J Nucl Med.

[22] Lin WY, Hsu WH, Lin KH, Wang SJ (2012). Role of preoperative PET-CT in assessing mediastinal and hilar lymph node status in early stage lung cancer. J Chin Med Assoc.

[23] Nakanishi K, Nakamura S, Sugiyama T, Kadomatsu Y, Ueno H, Goto M (2021). Diagnostic utility of metabolic parameters on FDG PET/CT for lymph node metastasis in patients with cN2 non-small cell lung cancer. BMC Cancer.

[24] Spadafora M, Pace L, Evangelista L, Mansi L, Del Prete F, Saladini G (2018). Risk-related (18)F-FDG PET/CT and new diagnostic strategies in patients with solitary pulmonary nodule: the ITALIAN multicenter trial. Eur J Nucl Med Mol Imaging.

[25] Redondo-Cerezo E, Martínez-Cara JG, Jiménez-Rosales R, Valverde-López F, Caballero-Mateos A, Jérvez-Puente P (2017). Endoscopic ultrasound in gastric cancer staging before and after neoadjuvant chemotherapy a comparison with PET-CT in a clinical series. United Eur Gastroenterol J.

[26] Zhou X, Wang S, Xu X, Meng X, Zhang H, Zhang A (2022). Higher accuracy of [(68) Ga]Ga-DOTA-FAPI-04 PET/CT comparing with 2-[(18)F]FDG PET/CT in clinical staging of NSCLC. Eur J Nucl Med Mol Imaging.

[27] Wang L, Tang G (2022). Comparison of (68)Ga-FAPI and (18)F-FDG PET/CT in the evaluation of advanced lung cancer. Radiolog.

[28] Stahlie EHA, van der Hiel B, Bruining A, van de Wiel B, Schrage YM, Wouters M (2021). The value of lymph node ultrasound and whole body (18)F-FDG PET/CT in stage IIB/C melanoma patients prior to SLNB. Eur J Surg Oncol.

[29] Calais J, Mona CE (2021). Will FAPI PET/CT replace FDG PET/CT in the next decade? point-an important diagnostic, phenotypic, and biomarker role. AJR Am J Roentgenol.

[30] Rovera C, Berestjuk I,Lecacheur M. 2022. Secretion of IL1 by Dedifferentiated Melanoma Cells Inhibits JAK1-STAT3-Driven Actomyosin Contractility of Lymph Node Fibroblastic Reticular Cells. 10.1158/0008-5472.CAN-21-0501 10.1158/0008-5472.CAN-21-0501 35502542

[31] Nizri E, Bar-David S, Aizic A, Sternbach N, Lahat G, Wolf I (2019). Desmoplasia in lymph node metastasis of pancreatic adenocarcinoma reveals activation of cancer-associated fibroblasts pattern and T-helper 2 immune cell infiltration. Pancreas.

[32] Itou RA, Uyama N, Hirota S, Kawada N, Wu S, Miyashita S (2019). Immunohistochemical characterization of cancer-associated fibroblasts at the primary sites and in the metastatic lymph nodes of human intrahepatic cholangiocarcinoma. Hum Pathol.

[33] Vilmann P, Clementsen PF, Colella S, Siemsen M, De Leyn P, Dumonceau JM (2015). Combined endobronchial and esophageal endosonography for the diagnosis and staging of lung cancer: european society of gastrointestinal endoscopy (ESGE) guideline, in cooperation with the european respiratory society (ERS) and the European society of thoracic surgeons (ESTS). Endoscopy.

